# SNPs in toll-like receptor (*TLR*) genes as new genetic alterations associated with congenital toxoplasmosis?

**DOI:** 10.1007/s10096-012-1763-y

**Published:** 2012-11-17

**Authors:** W. Wujcicka, J. Wilczyński, D. Nowakowska

**Affiliations:** Department of Fetal-Maternal Medicine and Gynecology, Polish Mother’s Memorial Hospital Research Institute, 281/289 Rzgowska Street, Lodz, 93-338 Poland

## Abstract

Nearly 40 % of pregnant women are infected with *Toxoplasma gondii*. Primary infections in pregnant women result, in approximately 30–50 % of patients, in transmission of *T. gondii* through the placenta to the fetus and then in congenital infections with severe, sometimes fatal course. Studies still do not provide sufficient data on the genetic bases of the immunity in fetuses, newborns, and infants with congenital toxoplasmosis. Previous research showed the contribution of toll-like receptors (TLRs) to non-specific immunity against *T. gondii* invasion, observed in *T. gondii*-infected animals, especially mice. So far, the activity of TLRs in defense against *T. gondii* infections was observed particularly for TLR2, TLR4, and TLR9 molecules. Differential TLR activity associates with both cell types, including a variety of placental cells and stage of pregnancy. Several single-nucleotide polymorphisms (SNPs) residing in three genes encoding these receptors were reported as significant genetic modifications of *TLRs* associated with different pregnancy disorders. Despite those data, genetic alterations of *TLRs* which have contributed to innate immune response against *T. gondii* infections are still not precisely described. In this article, we present reasons for the research of the plausible role of SNPs residing in *TLR2*, *TLR4*, and *TLR9* genes in congenital toxoplasmosis development.

## TLRs contribute to *Toxoplasma gondii* infections

Toxoplasmosis is one of the most frequent pregnancy infections transmitted from mother to child and the major cause of perinatal morbidity and mortality [1–3]. In Poland, nearly 40 % of pregnant women are infected with *Toxoplasma gondii* [4, 5]. Meiosis occurred in oocysts of *T. gondii* and resulted in the production of many different genotypes of parasitic sporozoites; however, three predominant distinct clonal lines (virulent strain I and non-virulent strains II and III) were found in Europe and North America [1, 6, 7]. Primary infections are dangerous in pregnant women, occurring usually asymptomatically, resulting, in approximately 30–50 % of patients, in transmission of *T. gondii* through the placenta to the fetus. As a result, these infections cause, in the fetuses and newborns, with immature immune systems, congenital infections with very severe, sometimes fatal course [3, 5, 8]. So far, extensive investigations aimed to describe mechanisms of congenital infections and related immune responses. However, current studies do not provide sufficient data on the associated alterations and genetic background of the innate immunity to *T. gondii* occurring in newborns and infants congenitally infected with this parasite. Three of ten human toll-like receptors (TLRs), TLR2, TLR4, and TLR9, have been reported to play roles in the recognition of *T. gondii*, course of infections with this parasite, and also in pregnancy progression and disorders.

**Fig. 1:**
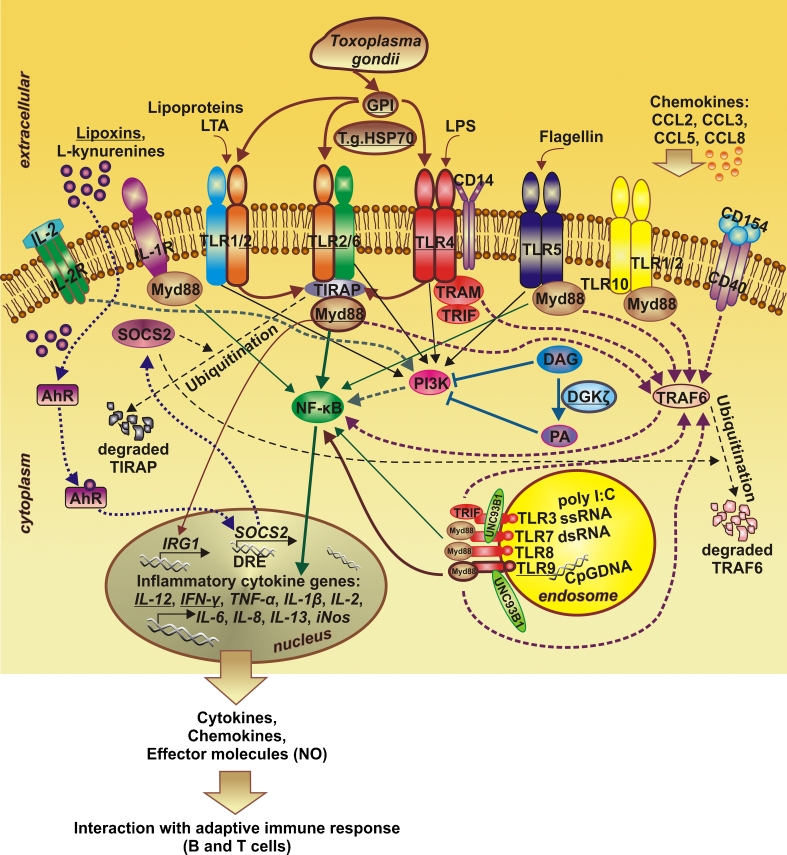
Toll-like receptor (TLR) signaling pathways involved in immune response to *Toxoplasma gondii* infections. Molecules considered to be crucial during *T. gondii* infection are underlined and surrounded by a thick continuous line

So far, studies clearly confirmed the essential role of TLR/MyD88 signaling in non-specific antimicrobial immune response to *T. gondii* infections. Many previous works reported the crucial role of IL-12 and of IFN-γ, the major regulators of IFN-γ production, by NK and T cells and cell-mediated immunity to *T. gondii*, respectively ([9, 10], reviewed in [11, 12]). The high expression levels of these two cytokines are stimulated after primary infection with *T. gondii* [11]. In mice, TLR11 was reported as the main regulator of IL-12 production following *T. gondii* infection ([11, 13], reviewed in [14]). However, TLR11 in humans is only a non-functional pseudogene [11, 15]. Hence, in this study, we described the role of other receptors from the TLR family in the defense against *T. gondii*. We suppose that genetic modifications, especially of *TLR2*, *TLR4*, and *TLR9*, might play a plausible role in congenital toxoplasmosis development. To argue the major role of TLRs in innate immune response to *T. gondii*, we described the most recent studies showing their involvement in immunity to this parasite. Figure 1 illustrates the activities and interactions of TLRs and other molecules reported to be involved in the immune response to *T. gondii* infection.

The latest study performed to describe the method of action of lipoxin- and L-kynurenine-induced SOCS2 in the pro-inflammatory response during infection also with *T. gondii* showed the engagement of both mediators in the inhibition of TLR/MyD88, TLR/TRIF, IL-1R/MyD88, and CD40/CD154 signaling pathways [16] (Fig. 1). Other studies reported that TLR response to *T. gondii* is promoted by the ζ isoform of the diacylglycerol (DAG) kinases family (DGKζ), expressed in macrophages (MΦ) and dendritic cells (DC) via a pathway involving the inhibition of PI3K [17] (Fig. 1). DGKζ deficiency resulted in impaired IL12 and TNF-α production after in vitro and in vivo TLR stimulation, elevated resistance to endotoxin shock, and increased susceptibility to *T. gondii* infection [17]. Studies which aimed to describe the role of DC in the immune response to *T. gondii* showed that mice with targeted inactivation of MyD88 in these cells, but not in macrophages or neutrophils, were highly susceptible to the *T. gondii* infection [18]. Subsequent results indicated the crucial role of TLR recognition by DC in the creation of a rapid type 1 innate immunity to *T. gondii* and to prevent acute mortality [18]. Murine CD11c+Gr-1+ DC infected with *T. gondii*, in contrast with non-infected cells, had no ability for ex vivo TLR stimulation [19]. Then, the observed results suggested that plasmacytoid DCs (pDCs) were utilized by *T. gondii* as Trojan horses in the early infection, with suppressed cytokine effector function, and exploited to disseminate the parasite within the host [19]. Earlier studies also reported the important cell-intrinsic role of MyD88 in the activation of neutrophils, macrophages, DC, and T cells during immunity to *T. gondii* [11, 12]. However, the function of MyD88 in neutrophils, macrophages, and monocytes has to be determined in detail. MyD88^−/−^ mice orally infected with *T. gondii* failed to control the parasite and succumbed within 2 weeks of infection, but the i.p. vaccinations of these mice with avirulent *T. gondii* uracil auxotroph induced strong IFN responses and protective immunity to the invasion of high-virulence *T. gondii* strains [20]. Hence, the results of this study confirmed that MyD88 is required to control *T. gondii* infection, but the adaptive immunity might be induced without the involvement of TLR adaptor molecule [20].

As MyD88 might be activated by almost all TLRs except TLR3, recent investigations also aimed to identify particular receptors crucial for the initial recognition of *T. gondii*. One of the latest studies showed that single nucleotide-sensing TLR3, TLR7, and TLR9 have no function in controlling the initial activation of innate immune response and host resistance to *T. gondii* infection in the 3d mice with a non-functional UNC93B1, a critical mediator of the translocation of the three investigated TLRs from ER to endolysosomes [21] (Fig. 1). However, 3d mice were extremely susceptible to infection with *T. gondii*. Therefore, the likely combined action of nucleotide-sensing TLR3, TLR7, and TLR9 in host defense against *T. gondii* was suggested [21]. 

Studies of 264 healthy Russian Karelian children performed to analyze the influence of the gene environment effect of *T. gondii*, *Helicobacter pylori*, CD14 −159C>T, and TLR4 +1896A>G polymorphisms on the total serum IgE showed no association between *T. gondii* or *H. pylori* seropositivity, or CD14 and TLR4 polymorphisms with IgE levels [22]. However, the constructed multiway analysis of variance (ANOVA) model showed the influence of CD14 −159 allele T and *H. pylori* antibodies status on the serum total IgE [22].

## The role of TLR2, TLR4, and TLR9 in *Toxoplasma gondii* infections

Among TLR characteristics for human cells, the activity in sensing *T. gondii* was especially determined for TLR2, TLR4, and TLR9 molecules [23–26]. In the transcriptome-profiling study performed to identify genes synergistically up-regulated by IFN-γ and TNF in macrophages, several proinflammatory cytokines and also TLR agonists were reported to stimulate increased expression of newly identified immunoresponsive gene 1 (IRG1) transcript [27]. The expression of IRG1 transcript was up-regulated after the stimulation of TLR2, TLR4, and TLR9 by LTA, lipopolysaccharide (LPS), and CpG, respectively. As IRG1 expression was not regulated by TLR3 stimulated by poly I:C, which is not signaled via MyD88, the molecule involved in the signaling of TLR2, TLR4, and TLR9, the MyD88 molecule, was suggested to be required for the induction of IRG1 [27] (Fig. 1). In other work, TLR2-deficient mice infected with 300 cysts of *T. gondii* died within a period of 10 days; however, all of them survived when the parasite dose was lower [28]. In *T. gondii* infection cases, TLR2 participated in macrophages activation and regulation of parasite-induced NO production [28]. Another study showed that iNOS^−/−^ mice were able to control the acute *T. gondii* infection, in which additional effector mechanisms were possibly regulated by TLR2 [29]. This receptor was also reported to regulate the *T. gondii*-induced production of the neutrophil-attracting chemokine CCL2 [30]. Experiments with the vaccination of *T. gondii*-infected mice with *T. gondii*-derived heat shock protein 70 (*T.g.HSP70*) gene showed its involvement in DC activation and Th1 polarization observed at draining lymph nodes (dLN) from wild type and TLR2-deficient mice, but not TLR4-deficient individuals with B6 background [31]. Early Th1 polarization was induced at the dLN of mice by the *T.g.HSP70* gene vaccine through the TLR4/MyD88 signal pathway (Fig. 1). At an acute phase of toxoplasmosis, the *T.g.HSP70* gene vaccine also limited the copy number of *T. gondii* in the mesenteric LN of WT, TLR2-deficient, and TRIF-deficient mice, but for neither TLR4-deficient nor MyD88-deficient mice, indicating the involvement of TLR4 in the vaccine effect at an acute phase of infection. What is more, TLR4 had activity in the determination of the *T. gondii* cyst number of the brain at a chronic phase of toxoplasmosis, observed also at 8 and 12 weeks post-infection [31]. TLR4-mediated macrophage activation was observed during *T. gondii* and *Leishmania donovani* infection, although this response was differentially regulated by progesterone via the glucocorticoid and progesterone receptors [23]. Another study showed that purified *T. gondii* glycosylphosphatidylinositols (GPI) triggered TLR4 signaling pathways as well [24]. Mice lacking both TLR2 and TLR4, but not TLR2 alone, had completely abrogated production of TNF in the response of *T. gondii* GPI [24]. However, mice deficient in TLR4 were only a little more susceptible to the *T. gondii* infection [24]. A significant role of TLR4 was reported in *T. gondii* infection observed in the small intestine [32]. TLR9-deficient mice infected orally with *T. gondii* were relatively resistant to the ileitis and had reduced Th1 response to the parasite [25, 26]. TLR9 deficiency in mice infected orally with *T. gondii* resulted in increased susceptibility to the infection and a 50 % reduction in IFN-γ production [26]. Then, the results proved that the TLR9 contribution coordinated both innate and adaptive immune response to *T. gondii*. However, the crucial indirect role in the stimulation of immunity to *T. gondii* is possibly characteristic for commensal gut bacteria rather than the parasite itself [25, 33].

## The activity of TLRs during pregnancy

The expression of *TLR* genes was observed in immune and also in non-immune cells such as trophoblasts, decidual cells, and amniotic epithelium [34–36]. The expression profile of *TLR* was associated with cell types, stage of pregnancy, and response to microorganisms ([34, 36–38], reviewed in [39, 40]). Studies performed for paraffin-embedded sections of endometrium and deciduas from first and second trimester elective terminations and third trimester normal deliveries showed alterations in the TLR4 expression levels between particular tissue samples [34]. Higher immunoreactivity of TLR4 observed in decidual cells compared to interstitial trophoblasts suggested maternally derived cells as the main protectors against Gram-negative bacteria and other harmful signals from severe inflammation associated with or without infection. Particularly, significantly higher TLR4 levels were observed in decidual cells of the first and third trimester compared to second trimester basalis, suggesting that an enhanced inflammatory environment occurred at the maternal–fetal interface in the initial stages and end of pregnancy compared to mid-pregnancy [34]. In turn, another study showed the presence of TLR2 and TLR4 proteins expressions from the one-cell stage through the blastocyst stage during murine embryo development [41]. In trophoblast stem cells, the expression of TLR2 and TLR6 but not TLR1 or TLR4 was observed [41]. At the plasma membrane, the expression of only TLR2 molecules was determined. The limited expression of TLRs on trophoblast cells suggested that early trophoblasts might be less able to protect embryos against pathogenic agents compared to differentiated trophoblast cells [41]. The obtained results are consistent with outcomes of another study, which indicated that TLR4 expression and related IL-8 secretion that occurred after LPS stimulation might be assigned, rather, to leukocytes, as it was no longer observed in trophoblast fraction purified from leukocytes [42]. The expression of different antimicrobial peptides and proteins was suggested to be characteristic for various placental cells and resulted from the cooperation between leukocytes and cells of embryonic origin [42]. Functional screening tests of TLRs in human amniotic epithelial cells showed that the expression of TLR5, TLR6/2, and TLR4 occurred in these cells [35]. The activation of TLR5 and TLR6/2 stimulated both IL-6 and IL-8 production and the NF-κB signaling pathway, whereas TLR4 activity was associated with reduced viability of analyzed cells and their apoptosis. Hence, differential immune response mediated via specific TLRs might even lead to preterm birth [35]. Other data suggested a contribution of TLR ligands to the direct activation of decidual NK cells and also NK cell lines observed in the presence of proinflammatory cytokines [43]. In human decidual mononuclear cells (DMNC), LPS, known as the TLR4 ligand, induced IFN-γ and TNF-α production, from which the former was IL-2- and IL-12-dependent, while the latter was independent [43]. Hence, further studies were suggested to be necessary to determine the mechanisms driving the synergistic effect of TLR and cytokine signaling [43]. Research of cultured cells of term placenta, including cytotrophoblast- and syncytiotrophoblast-rich cells, showed the expression of *TLR2*, *TLR3*, *TLR4*, *TLR5*, *TLR6*, and *TLR9* genes [36]. In first-trimester primary trophoblasts and trophoblast cell lines, the expression of *TLR1*, *TLR2*, *TLR3*, and *TLR4*, but not *TLR6*, was observed [37, 44]. However, the last gene was expressed in third-trimester trophoblasts, which suggested its regulation in a temporal manner [36, 37]. The *TLR4* expression level was higher in the term compared to the first-trimester placentas [45]. Placentas from early pregnancy were reported as being less responsive to pathogen agents than term tissues. TLR2 and TLR4 transcripts were identified in villous cytotrophoblast and extravillous trophoblast, but not in syncytiotrophoblasts in the first-trimester placenta [37]. It also suggested the regulation of TLR in a spatial manner. Then, it seemed that pathogens might be dangerous to the fetus only when the TLR-negative outer trophoblast layer is interrupted and infecting agents enter either the placental villous or the decidual compartments [37, 44]. The level of TLR1–TLR10 transcripts was also investigated in term human placentas collected in the absence of labor (elective cesarean sections; ECS) and after the completion of labor (normal vaginal delivery; NVD) [38]. TLR1–TLR10 transcripts were observed in all placentas; however, NVD tissues produced higher levels of TNFA in response to TLR4 (LPS) and to TLR7/8 agonist (resiquimod) than ECS placenta explants. Significantly elevated levels of TLR2 and TLR5 correlated with labor. Hence, this data suggested the role of TLR in parturition [38]. Studies of mRNA expressions of *TLR2*, *TLR3*, *TLR4*, and *TLR9* in the uterus, cervix, and placenta of non-pregnant and across gestation CD-1 mice showed significantly elevated levels in pregnant uterine and cervical tissues [46]. The differential levels of TLR mRNA expressions were observed between the uterus, cervix, and placenta with significantly down-regulated *TLR4* gene expression in the placenta [46]. The data suggested that the innate immune system was an active and dynamic system during gestation, with the altered expression of TLRs at the maternal–fetal interface playing a crucial role in the pathogenesis of adverse pregnancy outcomes.

Despite data confirming the involvement of TLR2, TLR4, and TLR9 receptors in the non-specific immunity to *T. gondii*, their contribution to toxoplasmosis development, and function within pregnancy, detailed mechanisms driving the activity of these receptors in *T. gondii* congenital infections are not known. So far, previous research showed the participation of several single-nucleotide polymorphisms (SNPs) of *TLR2*, *TLR4*, and *TLR9* in various diseases and pregnancy disorders [47–49].

## SNPs in the *TLR2* gene associated with pregnancy disorders

In a group of 200 cord blood samples obtained from 72 atopic and 128 non-atopic mothers, 12 SNPs located in *TLR1*, *TLR2*, *TLR4*, *TLR6*, and *TLR10* were genotyped to describe their possible influence on T-regulatory cells required for keeping immune responses in balance and roles played in atopic diseases [50]. In neonates, the presence of AA genotype of the *TLR2* promoter rs4696480 SNP associated with the increased expression of *FOXP3* and Treg marker genes *GITR* and *LAG3*, and also the secretion of TH2 cytokines and TNF-α in case of maternal atopy, with Tregs diminished without maternal atopy. The occurrence of GG genotype of *TLR2* rs1898830 correlated with Treg marker genes decreasing with and increasing without maternal atopy [50]. The screening of common *TLR2* (2258 G>A) and co-segregating *TLR4* (1063A>G and 1363C>T) SNPs in 94 women with preeclampsia and 176 healthy pregnancy controls showed the associations of these SNPs with early-onset but not late-onset preeclampsia [47]. Then, three *TLR* SNPs lowering thresholds for early-onset and severe pregnancy disease, but not late-onset or mild pregnancy disease, was described [47]. In turn, a study of 288 vaginal samples obtained from 144 women during both the first and second trimesters of pregnancy performed to determine the correlation of 34 SNPs residing in nine genes involved in TLR-mediated and -related sensing and regulation of pathogens with the vaginal carriage of Gram-positive anaerobes *Gardnerella vaginalis* and *Atopobium vaginae* showed no association between gene polymorphisms and bacterial vaginosis (BV) [51]. No significant correlation was determined for *TLR2* −15607A>G, 1350T>C, 2258G>A, and 2029C>T SNPs [51]. The lack of a relationship between *TLR2* polymorphisms and BV occurrence is consistent with the results of another study, performed to determine the influence of four *TLR2* SNPs [rs18988 (−15607 A>G), rs4696483, rs7656411, and rs1337)] on the cervical levels of pro- and anti-inflammatory cytokines and their association with BV [52]. In another study, analyses of the relevance of polymorphisms located in genes associated with innate immunity performed for a large cohort of preterm very-low-birth and term-born infants and their mothers showed no significant correlation between the presence of *TLR2* 2258G>A SNP and the occurrence of intrauterine infections as the cause of preterm birth [53]. Another study also showed that comparable frequencies of the mutated 2258G allele of the *TLR2* gene occurred in both pregnant women with preterm labor and healthy individuals [54]. In turn, another investigation reported that infants with two polymorphic *TLR2* alleles (−16934TA/AA and 2258GA/AA) had, importantly, shorter gestational ages [55].

## SNPs in the *TLR4* gene associated with pregnancy disorders

For the *TLR4* gene, 1063A>G and 1363C>T SNPs were the most commonly studied. Real-time polymerase chain reaction (PCR) assays followed by high resolution melt (HRM) analysis, performed to simultaneously identify SNPs from *TLR4* (1063A>G) as well as other genes *IL6*, *IL1β*, and *IL12RB* involved in the immune response showed no association of these polymorphisms with preterm birth of infants in Montevideo, Uruguay [48]. However, in the same population, fetuses carrying the 1063A>G SNP were both severely premature and had premature rupture of membranes (PROM) at the same time [56]. In a Canadian cohort which included mainly preterm infants, this *TLR4* SNP occurred at a significantly lower frequency in infants without bronchopulmonary dysplasia (BPD) compared to those with diagnosed BPD. However, no significant association was observed between *TLR4* genotypes and prematurity [57]. In turn, the research of a Finnish population showed the presence of the 1063A>G SNP in both infants and mothers and its correlation with preterm labor [58]. The frequency of *TLR4* 1063A/G alleles and their association with preterm labor might be population-specific. It seems that further determination of the contribution of the 1063A>G SNP to preterm labor is required. In contrast to *TLR2* polymorphisms, two SNPs located in the *TLR4* sequence (rs1554973 or rs7856729) interacted significantly with *IL-1R2* rs485127 and correlated with the concentration of IL-1β in 188 African American and European American women [59]. The study showed both *TLR4* and *IL1-R2* genotypes as possible significant markers of cervical cytokines IL-1α or IL-1β concentrations and involvement in disparity in pregnancy outcomes. However, the observed associations were no longer significant after correction for multiple testing in European or African Americans [59]. An earlier study reported a genetic association between *TLR4* rs1554973 and the level of cervical cytokine IL-1β characteristic for European but not African Americans, which was more significant in women with BV compared to those without BV [52]. These results are consistent with the observation that selection on TLRs varies between human populations [60]. In another study, the carriage of *TLR4* rs1554973 SNP strongly associated with chorionic plate inflammation both in mothers and their singleton fetuses [61]. Four SNPs of *TLR4* [1063A>G (rs4986790), 1363C>T (rs4986791), rs6478317, rs10759932] were reported to affect T-cell regulation [50]. Two common 1063A>G (rs4986790) and 1363C>T *TLR4* SNPs correlated with increased IL-13 secretion in core blood mononuclear cells (CBMC) obtained from umbilical veins after delivery and stimulated with *Dermatophagoides pteronyssinus*. CBMC with *TLR4* rs6478317 (GG) had higher IL-12 secretion observed after stimulation with lipid A (LpA). 1063A>G (rs4986790) and 1363C>T *TLR4* SNPs were associated with a higher risk for atopic dermatitis [50]. Similarly to *TLR2* SNPs, no *TLR4* polymorphisms (rs1927914, 1063A>G, 1363C>T) were associated with BV in 288 vaginal samples obtained from 144 women during both the first and second trimesters of pregnancy [51]. However, another study showed that the 1063A>G SNP, disturbing response to LPS, was correlated with an increase in the vaginal pH and levels of *Gardnerella vaginalis* and anaerobic Gram-negative bacteria [62]. In a study of the occurrence of the 1363C>T SNP in pregnant women with preterm labor and a control group, a lower frequency of heterozygous CT genotype and mutated T allele was observed in the study group [54]. Hence, this SNP might play a protective role against PROM in pregnant women. However, the proposed hypothesis has to be further investigated in a larger group of patients with PROM [54]. In *Plasmodium falciparum*-infected primiparous Ghanaian women, the 1063A>G SNP elevated the risk of low birth weight in term infants 6-fold and the risk of maternal anemia 5-fold [63]. The obtained results suggested a correlation of the 1063A>G SNP with a clinical picture of malaria during pregnancy. However, this mutation did not associate with preterm labor [63], whereas the 1363C>T SNP was, importantly, less common in women with BV compared with individuals without BV [64].

## SNPs in the *TLR9* gene associated with diseases and ocular toxoplasmosis

The accumulated data argued also for the association of different *TLR9* SNPs with a variety of disorders, along with toxoplasmic retinochoroiditis, cervical cancer, mother-to-child transmission (MTCT) of human immunodeficiency virus type 1 (HIV-1), increased risk of low birth weight in infants, risk of maternal anemia, and a clinical picture of malaria in pregnancy [49, 63, 65, 66]. The 1635A>G SNP residing in the *TLR9* gene was reported to correlate with toxoplasmic retinochoroiditis in Brazil [49]. In the studied population, ocular toxoplasmosis was associated with allele C at 1635A>G [odds ratio (OR) 7; 95 % confidence interval (CI) 1.6–30.8), which was at a frequency of 0.424 %, similar to that observed in European populations. The determined correlation suggested that direct interaction between *T. gondii* and TLR9 might trigger proinflammatory responses and, hence, led to severe pathologies such as ocular disease, associated with this infection in Brazil [49]. A study performed for the Polish population showed the association of *TLR9* 1635A>G and also rs187084 polymorphisms with cervical cancer and the possible significance of these SNPs as risk factors of cervical cancer development [65]. In turn, another work showed the role of the c.4-44G>A and c.1635A>G SNPs in MTCT of HIV-1 [66]. While neither of the two SNPs were associated with a risk of HIV-1 infection, the [G;G] haplotype correlated with a higher risk of MTCT of HIV-1 after adjustment for maternal viral load [66]. A study performed for *P. falciparum*-infected primiparous Ghanaian women reported a correlation of the −1486T>C SNP residing in the *TLR9* gene, just as in the case of the 1063A>G SNP in the *TLR4* gene, with an elevated risk of low birth weight in term infants and the risk of maternal anemia and, therefore, with a clinical picture of malaria in pregnancy [63].

## Concluding remarks

It is important to investigate molecular mechanisms involved in *Toxoplasma gondii* congenital infections more precisely. It seems necessary to identify, in clinical specimens such as blood, amniotic fluid, cerebrospinal fluid, and the placenta, the genetic alterations of *TLR2*, *TLR4*, and *TLR9* genes accompanying the development of these infections and to describe the clinical significance of single-nucleotide polymorphisms (SNPs) residing in these genes in regard to congenital toxoplasmosis. *TLR* genes were suggested to play roles in toxoplasmosis development, progression of pregnancy, and various disorders of gestation. One possible modification influencing gene function, which was also confirmed in case of *TLR2*, *TLR4*, and *TLR9* genes in accordance to different diseases and pregnancy disorders, is the occurrence of SNPs within the genetic sequence. Therefore, the detailed description of the occurrence and the role of SNPs located in these three genes of the *TLR* family might pose a significant direction of research to determine new genetic alterations involved in immunity against *T. gondii* infections in pregnant women, their fetuses, and newborns.
